# Paternal Alcohol Exposure Reduces Alcohol Drinking and Increases Behavioral Sensitivity to Alcohol Selectively in Male Offspring

**DOI:** 10.1371/journal.pone.0099078

**Published:** 2014-06-04

**Authors:** Andrey Finegersh, Gregg E. Homanics

**Affiliations:** Departments of Anesthesiology and Pharmacology & Chemical Biology, University of Pittsburgh, Pittsburgh, Pennsylvania, United States of America; University of Queensland, Australia

## Abstract

Alcohol use disorder (AUD) is heritable, but the genetic basis for this disease remains poorly understood. Although numerous gene variants have been associated with AUD, these variants account for only a small fraction of the total risk. The idea of inheritance of acquired characteristics, i.e. “epigenetic inheritance,” is re-emerging as a proven adjunct to traditional modes of genetic inheritance. We hypothesized that alcohol drinking and neurobiological sensitivity to alcohol are influenced by ancestral alcohol exposure. To test this hypothesis, we exposed male mice to chronic vapor ethanol or control conditions, mated them to ethanol-naïve females, and tested adult offspring for ethanol drinking, ethanol-induced behaviors, gene expression, and DNA methylation. We found that ethanol-sired male offspring had reduced ethanol preference and consumption, enhanced sensitivity to the anxiolytic and motor-enhancing effects of ethanol, and increased *Bdnf* expression in the ventral tegmental area (VTA) compared to control-sired male offspring. There were no differences among ethanol- and control-sired female offspring on these assays. Ethanol exposure also decreased DNA methylation at the *BdnfÆ*promoter of sire's germ cells and hypomethylation was maintained in the VTA of both male and female ethanol-sired offspring. Our findings show that paternal alcohol exposure is a previously unrecognized regulator of alcohol drinking and behavioral sensitivity to alcohol in male, but not female, offspring. Paternal alcohol exposure also induces epigenetic alterations (DNA hypomethylation) and gene expression changes that persist in the VTA of offspring. These results provide new insight into the inheritance and development of alcohol drinking behaviors.

## Introduction

Alcohol Use Disorder (AUD) is prevalent and contributes to substantial costs to both individuals and society. Alcohol consumption contributes to $223 billion in societal costs annually in the United States [1] and 3.8% of deaths worldwide [2]. Despite its tremendous impact on society, AUD has few available pharmacological treatments and a high rate of relapse.

Although the heritability of alcoholism is estimated to be ∼50% among men [3], the genetic basis for this disease is poorly understood despite considerable scientific investment. Like many other complex, polygenic diseases, DNA sequence variations have been found to be associated with risk of acquiring AUD [4], [5]; however, these variants account for a small fraction of the total risk [6]. Emerging evidence from several converging fields has reinvigorated the idea that inheritance of acquired characteristics, “epigenetic inheritance,” is an adjunct to traditional modes of genetic inheritance. In rodent studies in which genetics and environment can be rigorously controlled, it is now established that environmental perturbations can produce phenotypic (without genotypic) alterations in the subsequent 1–3+ generations that were themselves never exposed. For example, in isogenic rodents, exposure to stress [7], endocrine disruptors [8], high fat diet [9], low protein diet [10], and olfactory fear conditioning [11] all can result in phenotypic changes in subsequent generations. Of direct relevance to the study reported here is a growing literature on transgenerational effects of drugs of abuse. Adult offspring derived from dams exposed to morphine prior to conception displayed enhanced behavioral sensitivity to morphine and other behavioral alterations [12]. Male offspring of cocaine-exposed sires surprisingly displayed a cocaine-resistance phenotype [13]. Finally, prenatal exposure to ethanol (EtOH) was associated with transgenerational effects on POMC expression that was inherited through the male germ line [14].

A number of studies support the idea that parental EtOH exposure prior to mating can alter the phenotype of offspring. In rodents, paternal preconception EtOH exposure induced developmental abnormalities including altered organ weights including brain [15], [16], thickening of cortical layers [17], and decreased testosterone levels [18]. Paternal preconception EtOH exposure also induced numerous behavioral abnormalities, including decreased grooming behavior in response to novelty or water immersion [19], altered spatial learning [20], decreased novelty seeking [21], and decreased immobility in a forced swim test [22]. These changes did not appear to be related to stress and/or undernutrition associated with EtOH exposure.

The studies cited above led us to hypothesize that EtOH drinking behavior and neurobiological sensitivity to EtOH are due in part to paternal EtOH exposure prior to conception. To test this hypothesis in a model system that was free from confounding genetic and environmental influences, adult male mice were chronically exposed to EtOH (or control conditions) and subsequently mated to EtOH naïve females. Adult offspring were tested for EtOH drinking on the two bottle choice test and a range of EtOH-induced behaviors.

## Materials and Methods

### Animals

All experiments were approved by the Institutional Animal Care and Use Committee of the University of Pittsburgh and conducted in accordance with the National Institutes of Health Guidelines for the Care and Use of Laboratory Animals. Eight-week-old, EtOH-naïve, specific pathogen free C57BL/6J male and Strain 129Sv/ImJ female mice were purchased from the Jackson Laboratory and used to generate the F1 generation of hybrid offspring as described below. Mice were group-housed under 12 hour light/dark cycles and had ad libitum access to food and water.

### Paternal ethanol exposure

Vapor EtOH inhalation was used because it allows for ad libitum access to food and water, no stress-inducing injections or gavage, and animals remaining in their home cage. Two identical custom-built vapor chambers were used (16″×16″ ×24″ constructed from 0.5″ plexiglass) to deliver either room air or vaporized EtOH. Flow rate, vaporization temperature, and exposure time were optimized to achieve consistent blood EtOH concentrations (BEC) without the use of pyrazole. Room air was flowed into two heated Erlenmeyer flasks at a rate of 8 L/min; one flask received EtOH at a rate of ∼250 µl/min while the other flask received no EtOH. Air from the EtOH and control flasks flowed into separate chambers so that only one chamber received vaporized EtOH.

Male C57BL/6J mice were placed in vapor chambers from 08:00 to 16:00 for 5 consecutive days/week for 5 weeks. Five weeks of exposure was chosen because it represents a complete cycle of murine spermatogenesis [23]. Temperature of the chambers was monitored daily and averaged 78° F at the end of 8 h of exposure. Mice were weighed at the beginning of each week and blood was collected from the tail vein at the end of each week. Total EtOH in plasma was measured using an Analox EtOH analyzer (AM1, Analox Instruments, London, UK).

### Breeding scheme and offspring rearing

Immediately following the final day of exposure, male mice were removed from group housing and housed with two eight-week-old, EtOH-naïve Strain 129Sv/ImJ female mice. Strain 129Sv/ImJ females were chosen because they do not erase epigenetic marks at intracisternal A particles (IAPs) in offspring *in utero* while C57BL/6J females do erase these marks [24]. After 48 hours, males were removed from the female's cages. Strain 129xC57 F1 hybrid offspring were reared normally and weighed weekly beginning at 3 weeks of age. All offspring used in behavioral experiments were at least 8 weeks of age.

### Isolation of motile sperm DNA

Male mice were group housed and exposed to vapor EtOH or room air for an additional 3 days following mating. This additional exposure was done so that EtOH-induced epigenetic marks were not lost during this time period and that the effects of ethanol withdrawal on gene expression and epigenetic processes would not affect germ cells. Sixteen hours following exposure, the cauda epididymis was dissected from the testes and placed into 4 ml of 1% bovine serum albumin (BSA) in PBS. Sperm and DNA were extracted using a double swim up assay as previously described [25]. Briefly, several longitudinal cuts were made through the cauda epididymis using a scalpel and the tissue in 1% BSA was collected into a 15 ml conical tube. The tissue was incubated for 30 minutes at 37°C and the top 2 ml of liquid was collected into a new 15 ml conical tube, which was incubated for an additional 15 minutes at 37°C. The top 1 ml containing motile sperm was collected and used for analysis. Sperm were pelleted at 6000 rpm for 5 min at 4°C, resuspended in sperm lysis buffer with proteinase K [25], and incubated at 50°C overnight. DNA was extracted using a phenol-chloroform-isoamyl alcohol extraction and an EtOH precipitation and eluted in 100 µl TE buffer.

### Isolation of ventral tegmental area and medial prefrontal cortex RNA and DNA

Adult offspring were sacrificed by cervical dislocation and the brain extracted and placed into an ice cold adult mouse brain slicer matrix with 1 mm coronal section slice intervals (Zivic Instruments, Pittsburgh, PA). Razor blades were inserted starting at the rostral end through the midbrain. The ventral tegmental area (VTA) was defined on the first slice where the hippocampus wrapped around the midbrain as the region medial to the substantia nigra. The medial profrontal cortex (mPFC) was collected on the first two consecutive slices where the cortex was visible. These structures were collected, flash frozen in liquid nitrogen, and stored at -80°C. RNA and DNA were extracted using Trizol (Life Technologies, Carlsbad, CA) according to the manufacturer's protocol. RNA was further purified using the RNeasy mini kit with DNase digestion (Qiagen, Valencia, CA) and eluted in 30 µl water. DNA was eluted in 100 µl TE buffer.

### Bisulfite sequencing

Bisulfite treatment was carried out using the EZ DNA Methylation-Gold Kit (Zymo Research, Irvine, CA) according to the manufacturer's protocol. Briefly, 20 µl of DNA in TE buffer was treated with sodium bisulfite, desulphonated, and eluted in 10 µl of water. Bisulfite-treated DNA was used as a template for a nested PCR reaction. Primer sequences used were: *Bdnf* exon IXa promoter: Outside-F, 5′-ATA AAA AAA ATA ATA ACC ATC CTT TTC CTT ACT A-3′, Outside-R, 5′-ATT TAG GTA ATT TTT GTA TTT TTT TAG TAG AAA-3′, Inside-F, 5′-TAC CAT CCT TTT CCT TAC TAT TTT TAT TTC AT-3′, and Inside-R, 5′-GAG TAG AGG AGG TTT TAA AGG TAT TTG-3′; *IG* DMR: Outside-F, 5′-TTA AGG TAT TTT TTA TTG ATA AAA TAA TGT AGT TT-3′, Outside-R, 5′-CCT ACT CTAT AAT ACC CTA TAT AAT TAT ACC ATA A-3′, Inside-F, 5′-TTA GGA GTT AAG GAA AAG AAA GAA ATA GTA TAG T-3′, Inside-R, 5′-TAT ACA CAA AAA TAT ATC TAT ATA ACA CCA TAC AA-3′. PCR conditions for the outside reaction were: 4 min at 94°C, 2 min at 55°C, and 2 min at 72°C repeated once then 1 min at 94°C, 2 min at 55°C, and 2 min at 72°C repeated 35 times. The outside reaction was used as a template for the inside reaction, whose conditions were: 4 min at 94°C, then 1 min at 94°C, 2 min at 55°C, and 2 min at 72°C repeated 35 times, then 7 min at 72°C. The inside PCR reaction was run on a 1.2% agarose gel and the 320 bp product excised and gel purified using the Purelink Quick Gel Extraction kit (Life Technologies) according to the manufacturer's protocol. Gel-purified PCR products were cloned into a TOPO TA vector (Life Technologies) according to the manufacturer's protocol and transformed into TOP10 competent cells (Life Technologies). Cells were plated onto LB agar plates with ampicillin. Individual colonies were selected and grown overnight separately in LB with ampicillin, then plasmids were purified using the QIAprep Spin Miniprep Kit (Qiagen) according to the manufacturer's protocol.

Plasmids were checked for the presence of a 320 bp insert using an EcoRI digestion. Those with an insert were sent for sequencing (Genewiz, South Plainfield, NJ). After sequencing, trace files and the original BDNF exon IXa CpG island or *IG* DMR sequence were loaded into CpGviewer, an automated bisulfite sequencing analysis program that detects the methylation status of potentially methylated cytosines [26], and results were used for analysis. We performed at least two separate bisulfite treatments for each animal's tissue and at least four animals per group were used for analysis.

### Real-time Quantitative PCR (RT-qPCR)

300 ng of RNA was converted into cDNA using reverse transcriptase (RT) (Bio-Rad, Hercules, CA). Reactions were carried out in duplicate for each gene. SYBR green fluorescent master mix (Bio-Rad) was added to each well and visualized using a Bio-Rad iCycler. All primers were optimized for 90% to 110% efficiency at the following conditions: 10 min at 95°C followed by 40 cycles of 30 s at 95°C, 1 min at 60°C, and 30 s at 72°C. Primer sequences used were *β-actin*, F: 5′-TCA TGA AGT GTG ACG TTG ACA TCC GT-3′ and R: 5′-CCT AGA AGC ATT TGC GGT GCA CGA TG-3′, *Bdnf exon IXa*, F: 5′- AGC CTC CTC TAC TCT TTC TGC TG-3′ and R: 5′-GTG CCT TTT GTC TAT GCC CCT G-3′, *Bdnf exon IV*, F: 5′-CAG GAG TAC ATA TCG GCC ACC A-3′ and R: 5′-GTA GGC CAA GTT GCC TTG TCC G-3′, *Dlk1*, F: 5′-GGC CAT CGT CTT TCT CAA CA-3′ and R: 5′- ATC CTC ATC ACC AGC CTC CT-3′. Threshold cycle (Ct) values were calculated for each well and duplicate values averaged. The difference between specific genes and *β-actin* (ΔCt) was calculated for each animal and normalized to the average of room air sired offspring (ΔΔCt). Fold change over room air sired offspring was calculated for each animal using the following formula: 2^-ΔΔCt^.

### Two bottle choice

The two bottle choice test was used to assess the effect of paternal EtOH exposure on EtOH preference and consumption. Mice were acclimated to individual housing with food available ad libitum for one week. After one week, water bottles were replaced with two modified 25 ml polystyrene serological pipets (Thermo Fisher, Waltham, MA) fitted with ball bearing sipper tubes and filled with drinking water. Every 4 days, mice were weighed, total volume consumed measured by reading volume markers on the tubes, and tubes were removed, washed, and replaced. After 4 days of drinking water, tubes were replaced with one tube containing a 3% (w/v) EtOH solution and the other containing drinking water. This was immediately followed by 4 days each of a choice between 6%, then 9%, then 12%, and then 15% EtOH solutions and drinking water. The position of the EtOH tube was changed every 4 days, and there were no mice exhibiting a side preference for either tube position. A subset of mice was also tested for their preference for saccharin (0.033% and 0.066% w/v) then quinine hemisulfate (0.03 and 0.06 mM) following 15% EtOH solution. There was a 1 week washout period between EtOH, saccharin, and quinine tastants where both tubes contained water. Quantity of EtOH, saccharin, quinine, and water consumed was calculated for each mouse (g/kg/day or ml/kg/day) and preference was calculated as a ratio of solution consumed over total volume consumed. All solutions were also placed in an empty cage for 4 days to determine evaporation and spillage estimates, which were subtracted from the total volume consumed of each solution for each mouse.

### Elevated plus maze, open field, and accelerating rotarod

Offspring were tested for their performance on three consecutive behavioral tests after a single intraperitoneal injection with either 1 g/kg EtOH (0.02 ml/g body weight of 5% EtOH in 0.9% saline) or 0.02 ml/g 0.9% saline. Prior to injection, mice were individually housed with no food or water for 1 hour.

The elevated plus maze was used to assess the effect of paternal EtOH exposure on basal anxiety-like behavior and EtOH-induced anxiolysis. Ten minutes after injection, mice were place in the center of an elevated plus maze facing an open arm. Sessions were video recorded for 5 minutes and manually scored for number of arm entries and time spent in each arm; arm entries were defined as all four limbs within an arm. At the conclusion of the elevated plus maze assay, mice were returned to individual housing.

The open field test was used to assess the effect of paternal EtOH exposure on basal and EtOH-induced locomotor activity. Five minutes after the conclusion of the elevated plus maze (20 minutes post-injection), mice were placed in the corner of a 43.2×43.2×30.5 cm open field box with a white floor and clear plexiglass walls (Med Associates Inc., St. Albans, VT). The open field box was placed in a sound attenuating cubical (Med Associates Inc.) and illuminated by a 1W bulb and a small fan provided a low level of background noise. Movement was tracked using infrared beam sensors over a 10 minute trial.

The accelerating rotarod test was used to assess the effect of paternal EtOH exposure on basal motor coordination and EtOH-induced ataxia. Five minutes after the conclusion of the open field test (35 minutes post-injection), mice were habituated to the rotarod (Ugo Basile, Italy) for 30 seconds at 5 rpm. Then, the rotarod was accelerated from 5 to 50 rpm over 180 seconds. The amount of time mice remained on the accelerating rotarod was measured for 5 separate trials spaced 60 seconds apart.

### Ethanol metabolism

The rate of EtOH clearance was determined to assess the effect of paternal EtOH on EtOH metabolism. Mice were injected with 3.5 g/kg EtOH i.p. (0.02 ml/g of 17.5% EtOH in 0.9% saline). Blood was collected by tail nick at 60 minutes and 240 minutes post-injection. Total EtOH in plasma and standards was measured using an Analox EtOH analyzer.

### Statistical analysis

Behavioral experiments were analyzed using a Student's t-test, χ^2^ test, and two-way ANOVA with or without repeated measures where appropriate. For ANOVAs, post-hoc pairwise comparisons were made using the Fisher's LSD test. For analysis of DNA methylation, groups were analyzed using the Mann Whitney U test. For RT-qPCR data, groups were analyzed using a Student's t-test. Data from mice that differed by 2 SDs from the mean were considered outliers and excluded from analysis. Two data points were removed: one E-sired and one C-sired male on the 2 bottle choice experiment.

## Results

### Paternal ethanol exposure

**Figure 1:**
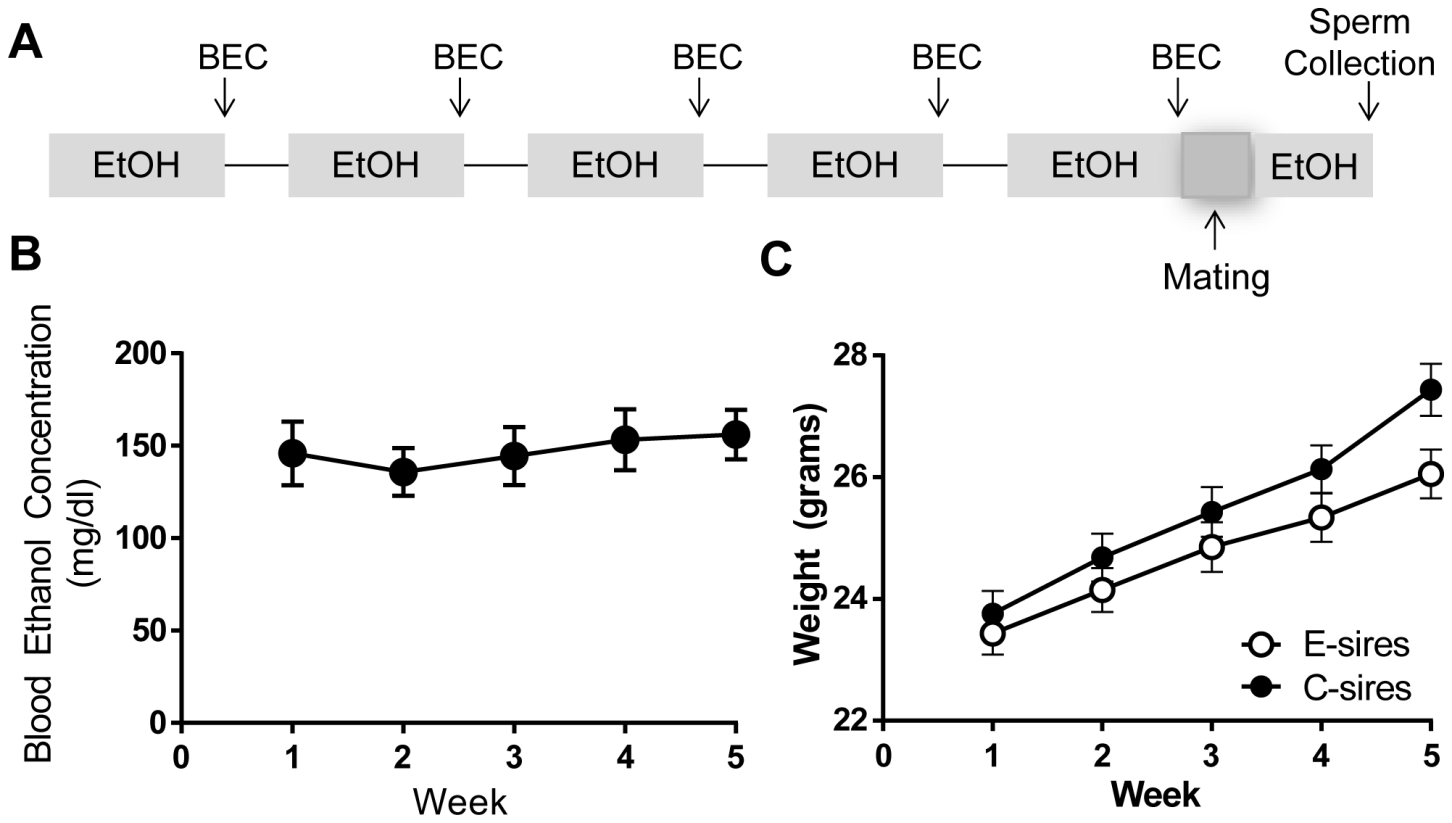
Chronic vapor ethanol exposure in sires. (A) 8-week-old C57BL/6J mice were exposed to EtOH vapor or room air for 8 hours/day, 5 days/week, for 5 weeks and immediately housed with 2 EtOH-naïve Strain 129Sv/ImJ females for 48 hours; after mating, they were re-exposed for 3 days and motile sperm was collected. (B) Blood EtOH concentrations showed limited variability across 5 weeks and averaged 147.1+/−7.52 mg/dl (mean ± SEM) (n = 25). (C) There was no difference in weight gain between EtOH (n = 25) and Room air (n = 27) exposed sires during the 5 weeks of exposure. Data presented as mean ± SEM bars.

Sires were exposed to vapor EtOH (E-sires) or room air (C-sires) for 8 hours per day, 5 days per week, for 5 weeks (Fig. 1A). This chronic intermittent EtOH exposure paradigm induces dependence [27] and increases voluntary EtOH drinking [28]. We subjected 3 separate cohorts to exposure and mating with 7–12 sires/group to generate 3 separate cohorts of offspring for behavioral experiments. BECs were measured on the final day of EtOH exposure each week and averaged 147.1+/−7.52 mg/dl (mean +/− SEM) in E-sires (Fig. 1B). Sires were weighed at the beginning of each week and there was a significant effect of time on sire weight over the course of five weeks (F_(4,200)_ = 54.50, p<0.0001) but no significant effect of treatment or interaction, indicating generally normal weight gain among E-sires (Fig. 1C).

### E-sired male offspring have increased weight after weaning

**Figure 2:**
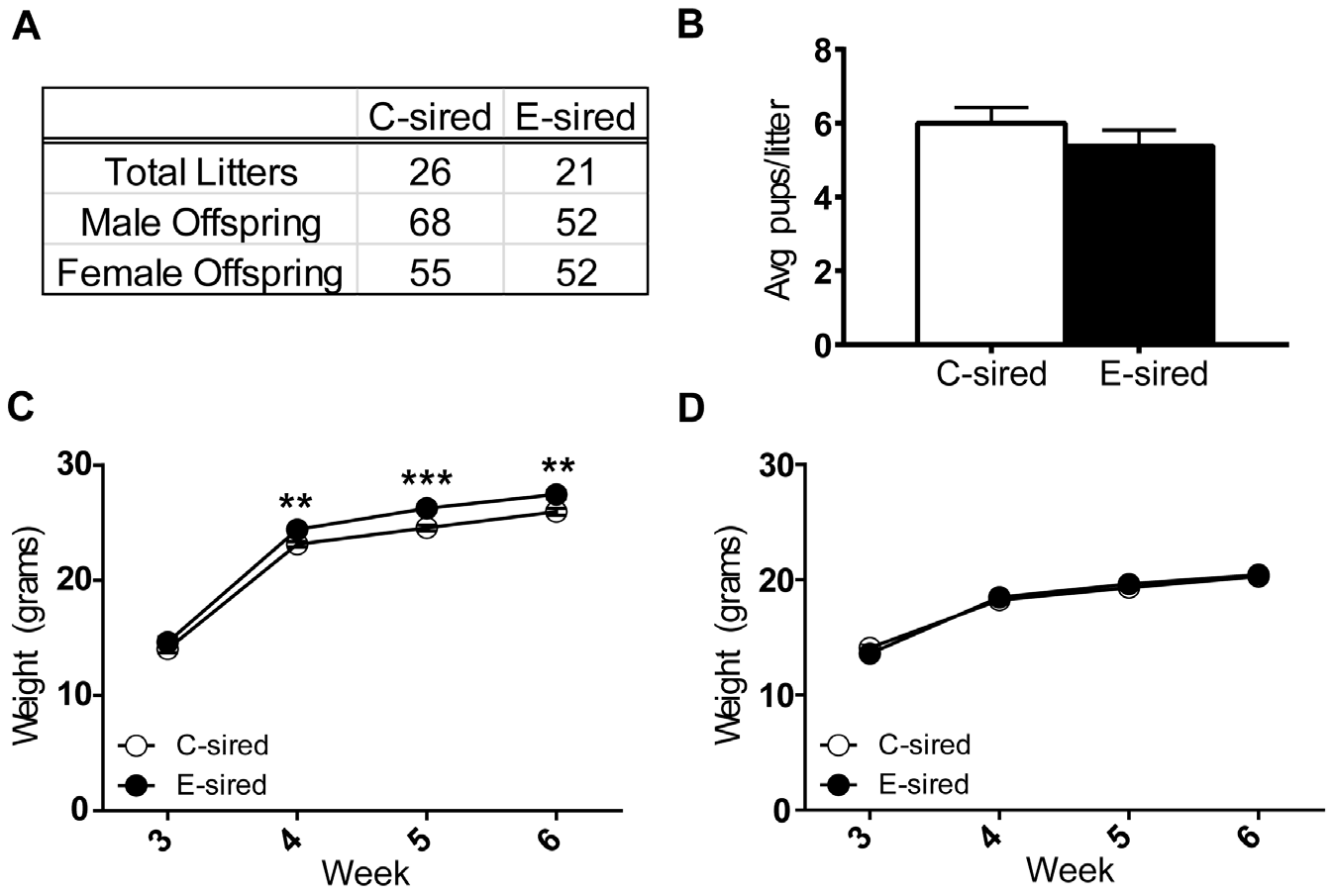
Comparison of E-sired and C-sired litters. (A) There were no differences in number of litters, number of male and female offspring, or (B) number of offspring per litter between E- and C-sires. (C) E-sired male offspring (n = 40) gained significantly more weight after weaning at 3 weeks and maintained increased weight through week 6 compared to C-sired male offspring (n = 61) (p<0.001). (D) There was no significant difference in weight between E- (n = 43) and C-sired (n = 45) female offspring. Data presented as mean ± SEM (Note: Error bars in C and D are obscured by the data points), **p<0.01, ***p<0.001.

Immediately following the final exposure, each sire was mated to 2 EtOH-naïve Strain 129Sv/ImJ females for 48 hours; there was no significant difference in the number of offspring sired or litter size from E-sires compared to C-sires (Fig. 2A–B). For body weight of male offspring, there was a significant effect of time (F_(3,297)_ = 900.7, p<0.0001) and sire exposure (F_(1,99)_ = 17.35, p<0.0001) but no interaction of time and treatment; post-hoc analysis revealed that E-sired male offspring weighed more than C-sired male offspring at 4 (p<0.01), 5 (p<0.001), and 6 weeks (p<0.01) of age (Fig. 2C). For body weight of female offspring, there was a significant effect of time (F_(3,258)_ = 508.3, p<0.0001), but no effect of sire exposure or interaction between time and treatment (Fig. 2D).

### E-sired male offspring consume less EtOH

**Figure 3:**
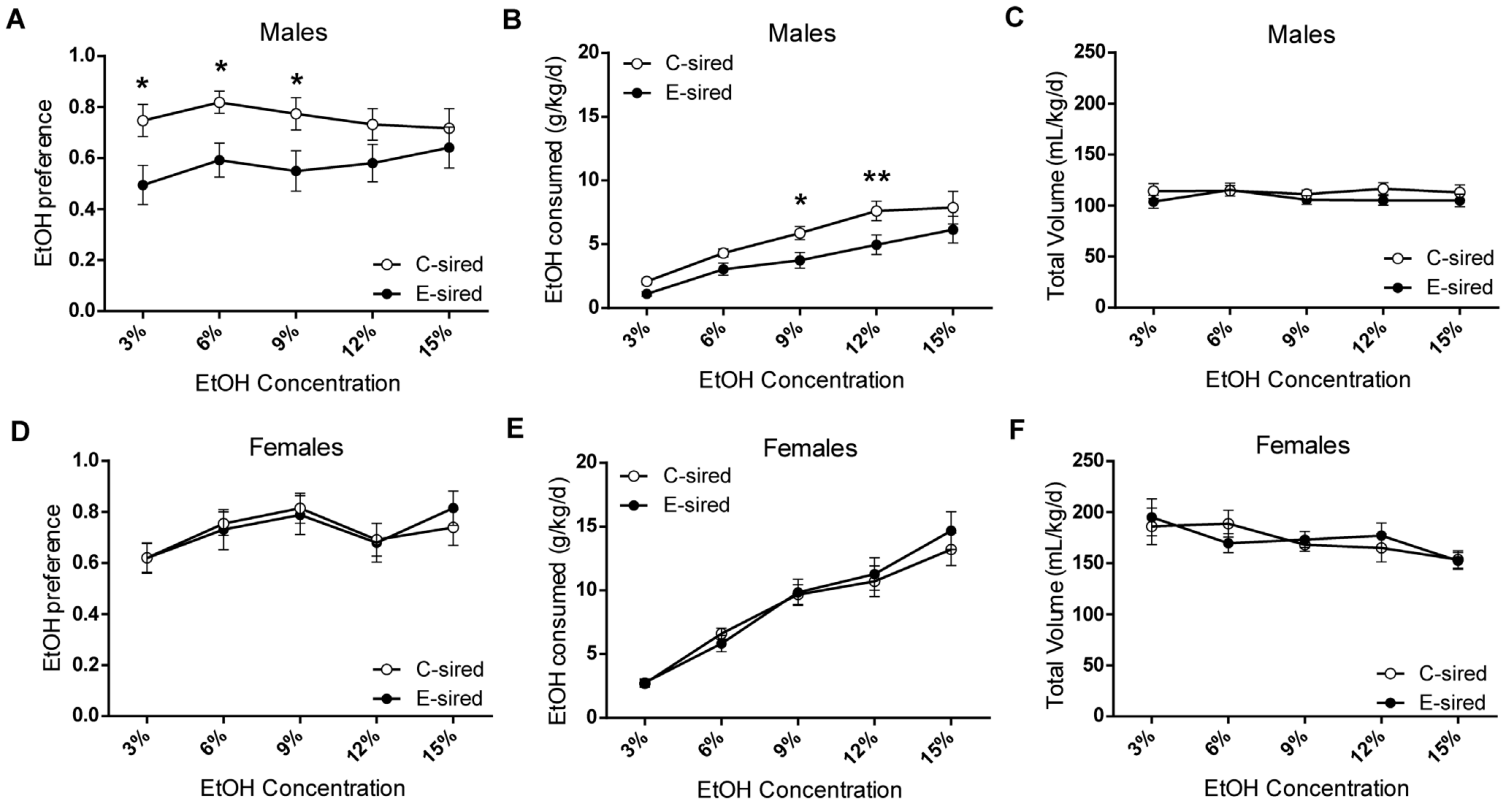
Offspring were tested for EtOH consumption vs. water on a 2 bottle, free choice drinking assay. (A) E-sired male offspring (n = 17) had significantly decreased preference for EtOH compared to C-sired male offspring (n = 17) as well as (B) decreased EtOH consumption and (C) no change in total volume consumption per body weight. There were no significant differences between E-sired female offspring (n = 12) and C-sired female offspring (n = 12) on (D) EtOH preference, (E) EtOH consumption, or (F) total volume consumed. Data presented as mean ± SEM, *p<0.05, **p<0.01.

Offspring were tested for their preference and consumption of EtOH versus water using a standard two bottle choice drinking paradigm. Offspring were tested sequentially for consumption of 3%, 6%, 9%, 12%, and 15% EtOH (w/v%) for 4 days each. For EtOH preference in male offspring, there was an effect for sire exposure (F_(1,32)_ = 7.22, p<0.05) but not for EtOH concentration and no interaction between sire exposure and concentration; post-hoc analysis revealed E-sired male offspring had significantly decreased preference for 3% (p<0.05), 6% (p<0.05), and 9% (p<0.05) EtOH solutions compared to C-sired male offspring (Fig. 3A). For EtOH consumption, there was a significant effect of sire exposure (F_(1,32)_ = 6.63, p<0.05) and EtOH concentration (F_(4,128)_ = 27.82, p<0.0001) but no interaction; post-hoc analysis revealed E-sired male offspring consumed significantly less of 9% (p<0.05) and 12% (p<0.01) EtOH solutions compared to C-sired male offspring (Fig. 3B). There was no effect of sire exposure, EtOH concentration, or interaction on total volume (EtOH + water) consumed (Fig 3C).

For females, there was an effect of EtOH concentration on preference (F(4,88) = 4.84, p<0.01; Fig. 3D), consumption (F(4,88) = 67.63, p<0.0001; Fig. 3E), and total volume consumed (F(4,88) = 4.01, p<0.01; Fig. 3F). However, there were no effects of sire exposure and no interaction of sire exposure with concentration on any parameter measured.

To investigate if any observed changes in EtOH drinking behavior were influenced by alterations in taste perception, mice were tested for preference of quinine or saccharin versus water in a similar two bottle choice assay. No effects of sire exposure, concentration, or interaction were observed for males consuming quinine (Fig. S1A) or saccharin (Fig. S1B) or for females consuming quinine (Fig. S1C) or saccharin (Fig. S1D).

### EtOH-induced anxiolysis and locomotor stimulation are enhanced in E-sired male offspring

**Figure 4:**
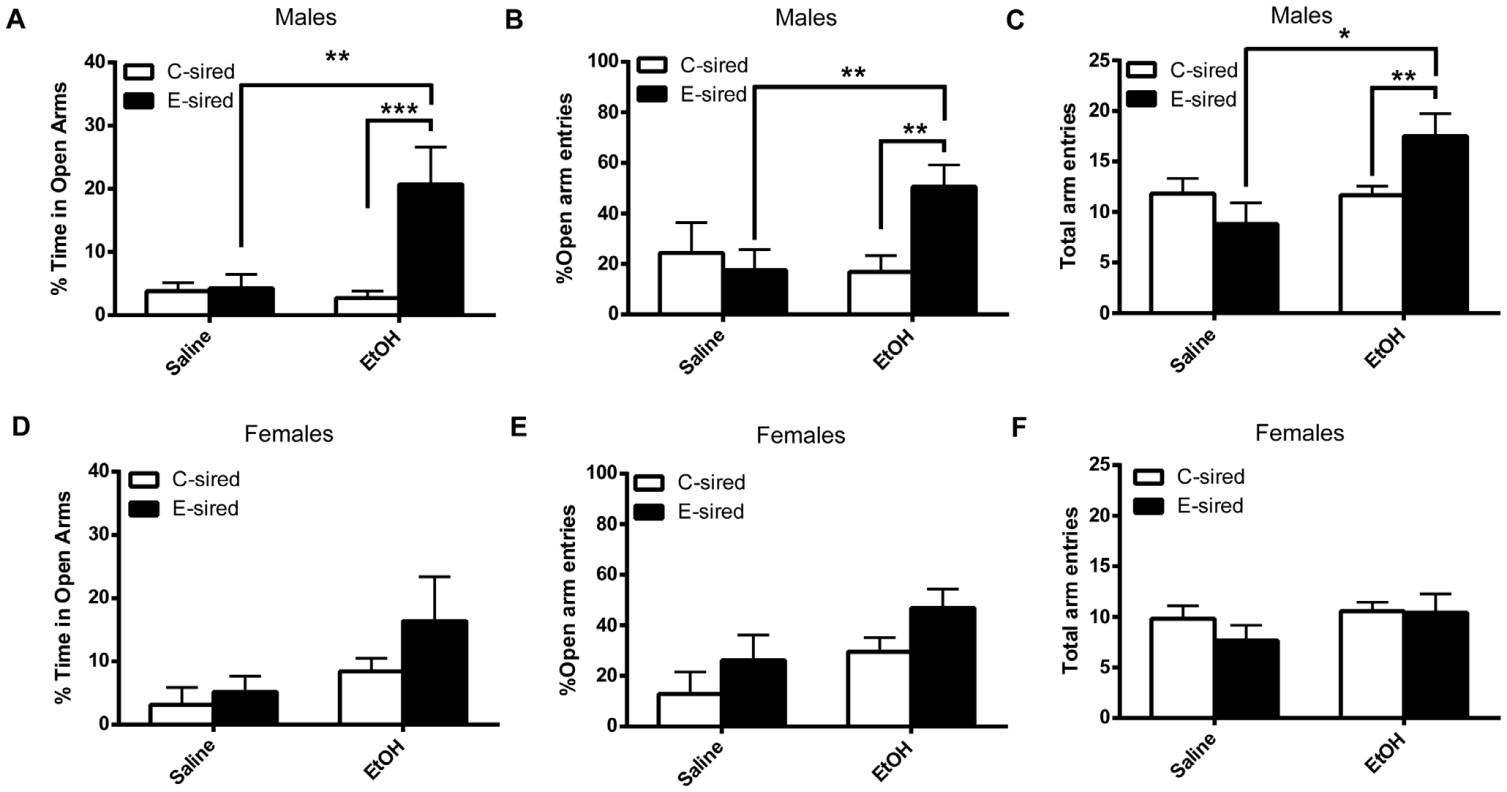
Offspring were tested for EtOH-induced anxiolysis by comparing performance on an elevated plus maze 10 minutes after i.p. injection of 1 g/kg EtOH or saline. (A) E-sired male offspring spend greater time in open arms after treatment with EtOH compared to C-sired male offspring and E-sired male offspring treated with saline; E-sired male offspring treated with EtOH also have (B) increased percent of open arm entries and (C) total arm entries relative to C-sired male offspring and E-sired male offspring treated with saline. There were no significant differences between E- and C-sired females treated with EtOH or saline on (D) time spent in open arms, (E) percent open arm entries, or (F) total arm entries. n = 6–7/group, data presented as mean ± SEM, *p<0.05, **p<0.01, ***p<0.001.

A separate group of EtOH naïve offspring were tested for performance on the elevated plus maze (EPM) 10 minutes after treatment with 1 g/kg EtOH or saline i.p. For male offspring, we observed a significant effect of treatment (F_(1,20)_ = 5.50, p<0.05) and sire exposure (F_(1,20)_ = 7.95, p<0.05) as well as a significant interaction between treatment and sire exposure (F_(1,20)_ = 7.21, p<0.05) on percent time spent in open arms; post-hoc analysis revealed E-sired male offspring treated with EtOH spent significantly more time in the open arms compared to those treated with saline (p<0.01) as well as C-sired male offspring treated with EtOH (p<0.001) (Fig. 4A). There was also a significant interaction between treatment and sire exposure (F_(1,20)_ = 4.97, p<0.05) on percent of open arm entries relative to total arm entries; post-hoc analysis revealed E-sired male offspring treated with EtOH had a significantly greater percent of open arm entries compared to those treated with saline (p<0.01) as well as C-sired male offspring treated with EtOH (p<0.01) (Fig. 4B). These results demonstrate that although basal levels of anxiety-like behavior did not differ between groups, male mice born to EtOH-exposed sires were more sensitive to the anxiolytic effect of EtOH.

There was also a significant effect of treatment (F_(1,20)_ = 5.91, p<0.05) but not sire exposure on total arm entries; post-hoc analysis revealed E-sired male offspring treated with EtOH made significantly more arm entries compared to those treated with saline (p<0.05) as well as C-sired male offspring treated with EtOH (p<0.01) (Fig. 4C). These results demonstrate that although basal levels of locomotor activity on the EPM did not differ between groups, male mice born to EtOH-exposed sires were more sensitive to the locomotor stimulatory effects of EtOH compared to male mice born to sires that were not exposed to EtOH.

For female offspring, there was no significant effect of treatment or sire exposure and no interactions for percent time spent in open arms (Fig. 4D). There was a significant effect of treatment (F_(1,22)_ = 4.56, p<0.05) but no effect of sire exposure or interaction on number of open arm entries (Fig 4E). There was no significant effect of treatment or sire exposure and no interactions for total arm entries (Fig. 4F).

### No changes in open field performance

Five minutes after the completion of the EPM (i.e., 20 minutes after treatment with either 1 g/kg EtOH or saline), the same mice were placed in an open field activity monitor and distance traveled was measured for 10 minutes. For male offspring, there was a trend for treatment (F_(1,24)_ = 3.152, p = 0.09) but no effect of sire exposure or interaction on distance traveled (Fig. S2A). For female offspring, there was no significant effect for treatment, sire exposure, or interaction on distance traveled (Figure S2B).

### E-sired male offspring have enhanced performance on accelerating rotarod

**Figure 5:**
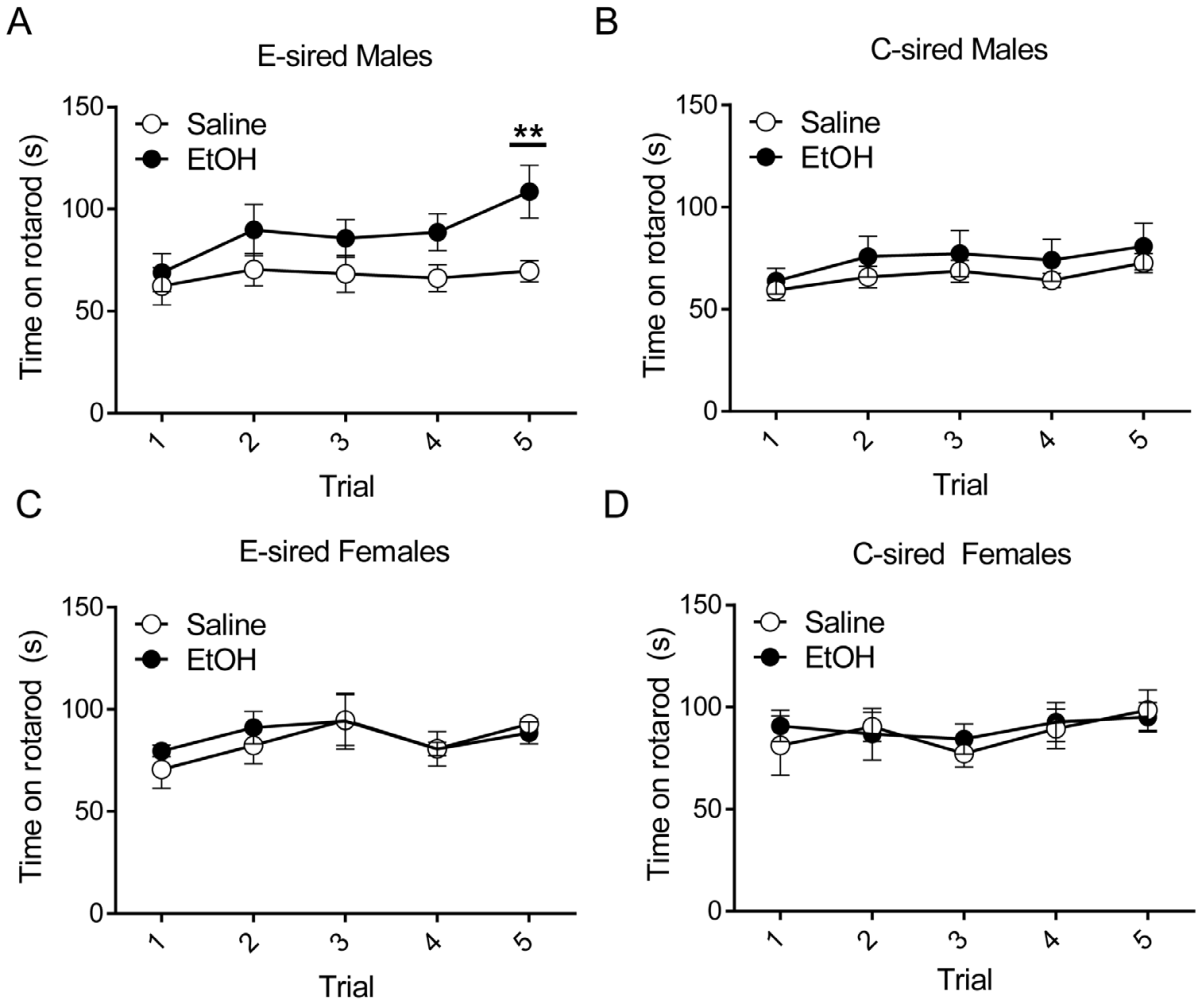
Offspring were tested for performance on an accelerating rotarod 35 minutes after i.p. injection of 1/kg EtOH or saline. (A) E-sired male offspring treated with EtOH performed significantly better on the 5^th^ trial compared to those treated with saline. There are no significant differences associated with EtOH treatment in (B) C-sired male offspring, (C) E-sired female offspring, or (D) C-sired female offspring. n = 7–8/group, data presented as mean ± SEM, *p<0.05.

Five minutes after completion of the open field test (i.e., 35 minutes after treatment with 1 g/kg EtOH or saline), the same mice were tested on five consecutive trials for their ability to remain on an accelerating rotarod. For E-sired male offspring, there was a significant effect of trial (F_(4,56)_ = 5.93, p<0.001), a trend of treatment (F_(1,14)_ = 3.21, p = 0.09), and a significant interaction between trial and treatment (F_(4,56)_ = 2.86, p<0.05); post-hoc analysis revealed E-sired male offspring treated with EtOH performed significantly better on the 5^th^ trial compared to those treated with saline (p<0.01) (Fig. 5A). For C-sired male offspring, there was a significant effect for trial (F_(4,56)_ = 3.48, p<0.05) but not treatment or interaction of trial with treatment (Fig 5B).

For E-sired and C-sired female offspring, there were no significant effects of trial, treatment, or interaction between trial and treatment (Fig. 4C,D).

### No change in EtOH Clearance

To ensure that differences observed on behavioral assays were not confounded by changes in EtOH pharmacokinetics, offspring were tested for the rate at which EtOH was cleared from the venous circulation. There was no significant effect of sire exposure on EtOH clearance in males or females (Fig. S3).

### E-sired male offspring have increased *Bdnf* exon IXa expression in the ventral tegmental area

**Figure 6:**
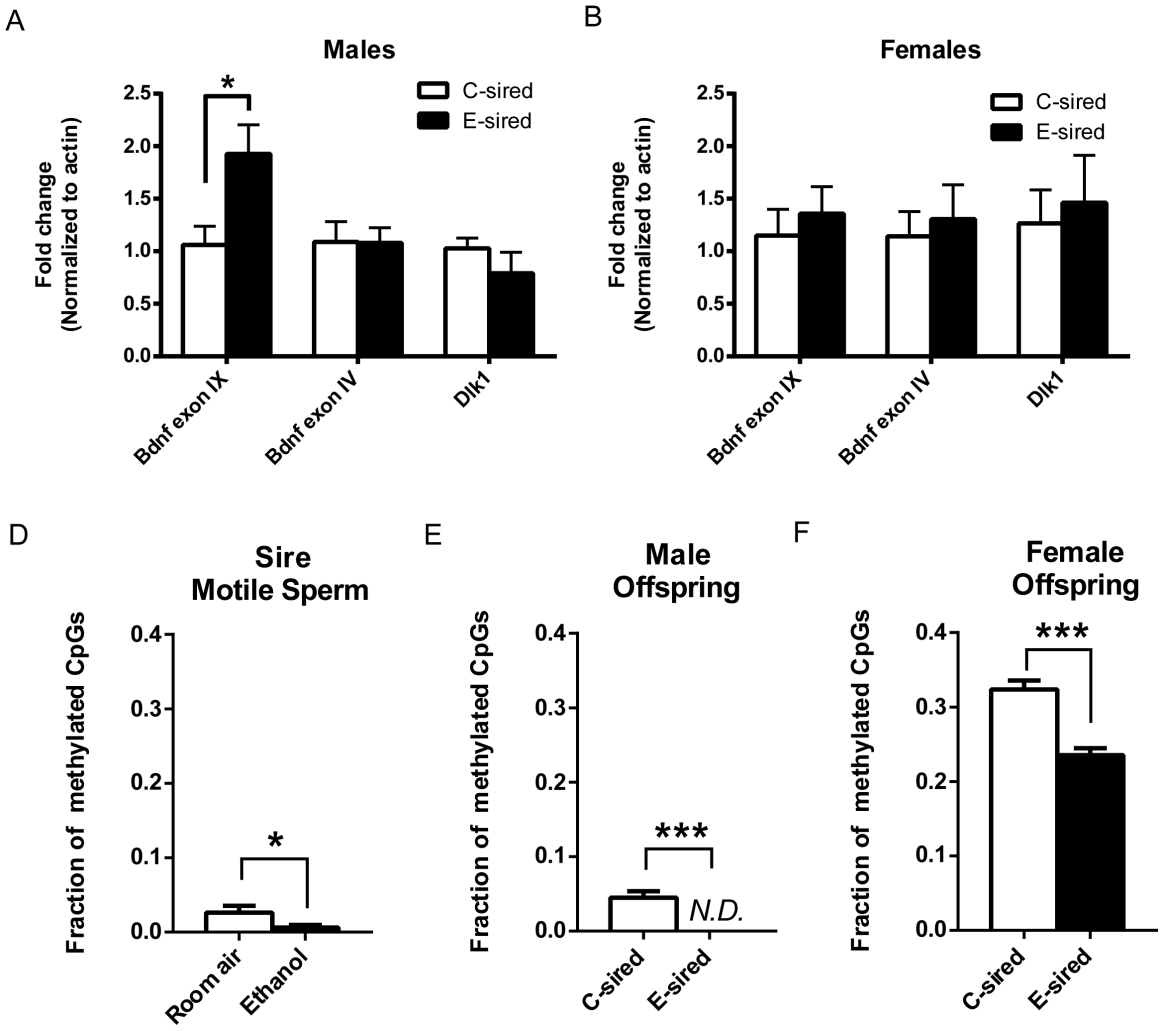
Expression of *Bdnf* and *Dlk1* were measured in the VTA of offspring and DNA methylation was measured at the *Bdnf* exon IXa promoter in motile sperm and offspring VTA. (A) E-sired male offspring had significantly increased expression of *Bdnf* exon IXa but not *Dlk1* or *Bdnf* exon IV in the VTA relative to C-sired males. (B) There were no significant differences in *Bdnf* or *Dlk1* expression between E- and C-sired females in the VTA. (C) EtOH-exposed sires had decreased DNA methylation at the *Bdnf* exon IXa promoter relative to Room air exposed sires. (D) Male and (E) female E-sired offspring had decreased DNA methylation at the *Bdnf* exon IXa promoter relative to C-sired offspring. N.D., none detected, n = 4–7/group, data presented as mean ± SEM, *p<0.05, ***p<0.001.

To determine if paternal EtOH exposure leads to lasting changes in brain gene expression in offspring, we examined *Bdnf* and *Dlk1* expression in offspring VTA and mPFC. *Bdnf* is a known regulator of EtOH drinking behavior [29]–[31] whose expression is up-regulated in male offspring of cocaine-exposed sires in the mPFC [13]. *Dlk1* is expressed from the paternal chromosome and is important for regulation of neurogenesis [32] and adipogenesis [33]. We chose to examine gene expression in the VTA because *Dlk1* is enriched in this region but has limited expression in other regions of the brain [34], [35]; *Bdnf* expression is also enriched at the VTA relative to other brain regions [36]. We chose to study *Bdnf* exon IXa expression because it is invariably expressed with all *Bdnf* mRNA sequences [37]. We found significantly increased expression of *Bdnf* exon IXa but not its activity-associated splice variant, *Bdnf* exon IV [38], or *Dlk1* in the VTA of E-sired male offspring (Fig. 6A). There were no differences in gene expression between E and C-sired female offspring in the VTA (Fig 6B). We also measured expression of *Bdnf* exons IV and IXa in the mPFC to study whether changes in expression generalized to another brain structure and to test potential similarities between E- and cocaine-sired male offspring. We did not find a significant difference in *Bdnf* expression between E- and C-sired offspring in the mPFC (Fig. S4).

### EtOH exposure decreases DNA methylation at the *Bdnf* exon IXa promoter in sires and their offspring

DNA methylation of the *Bdnf* exon IXa promoter regulates its expression [39], [40]; therefore, we hypothesized that EtOH exposure alters DNA methylation at the *Bdnf* exon IXa CpG island in sperm and that this epigenetic mark is inherited by male offspring and maintained in the VTA. We found that EtOH exposure significantly decreased DNA methylation in motile sperm (Fig. 6C, Fig. S5A) as well as in the VTA of E-sired male offspring (Fig. 6D, Fig. S5B). Surprisingly, E-sired female offspring also had decreased DNA methylation at the *Bdnf* exon IXa promoter (Fig. 6E, Fig. S4C). We also found hypomethylation of the intergenic (IG) differentially methylation region (DMR) in sperm (p<0.001) (Fig. S6), which regulates expression of *Dlk1* [41].

## Discussion

The results presented here raise the possibility that EtOH drinking behavior and sensitivity to the behavioral effects of EtOH may be epigenetically transmitted through the male lineage. We demonstrate in mice, where genotype effects can be rigorously controlled, that exposure of sires to EtOH prior to mating induces increased sensitivity to the anxiolytic and motor effects of EtOH and reduces EtOH preference and consumption exclusively in male offspring. E-sired male offspring also weighed significantly more than C-sired males after weaning, suggesting potential metabolic effects of paternal EtOH exposure. Consistent with our behavioral phenotype, we identified an increase in *Bdnf* expression in the VTA of male but not female offspring. Finally, we identified an EtOH-induced change to DNA methylation of the *Bdnf* promoter in motile sperm that persists in the brain of offspring. Remarkably, no behavioral or gene expression changes were observed in female offspring despite maintaining hypomethylation at the *Bdnf* promoter in the VTA. E-sired and C-sired offspring did not differ in consumption or preference of quinine or saccharin, and no differences were observed in the effects of EtOH on open field locomotor activity.

One particularly striking feature of the study presented here is how closely the results parallel that observed following paternal cocaine [13] and maternal preconception morphine [12], [42]. Vassoler et al. (2013) demonstrated that consumption of cocaine by sires imparted a cocaine resistant phenotype that was restricted to male offspring and females were phenotypically normal. The study also found changes in *Bdnf* expression in the brain in male offspring of cocaine-exposed sires. These similarities raise important questions about how drugs of abuse with distinct mechanisms of action produce a phenotype of drug resistance in male offspring. One explanation is that both are acting on a common pathway important for encoding and maintaining epigenetic marks in germ cells that ultimately control gene expression in brain of offspring. The work of Vassoler et al. (2013) implicates cocaine-induced changes in posttranslational histone modifications that ultimately influence brain expression of *Bdnf* in male, but not female offspring, as a causative contributor to the observed phenotype. We identified increased *Bdnf* exon IXa expression in only male offspring but found DNA methylation changes at the *Bdnf* exon IXa promoter in both male and female offspring. This result suggests that while EtOH-induced changes to DNA methylation may be inherited by both sexes, these changes are not the primary driver of the observed changes in *Bdnf* exon IXa expression. At present, the *Bdnf* exon IXa expression and behavioral changes are only correlative in nature. Additional studies are needed to establish a causal role of increased *Bdnf* exon IXa expression in the VTA on EtOH-induced behavioral changes observed in male offspring.

Our finding of decreased EtOH preference and consumption for E-sired male offspring is in apparent conflict with the familial nature of alcoholism observed in humans. Based on observations in human populations that sons of alcoholics have increased risk for alcoholism [43], one would predict that rodent E-sired offspring would also consume more EtOH. While the discrepancy between our findings and human studies could simply reflect species differences, it also may be due to differences in alcohol exposure. Whereas humans voluntarily consume high quantities of EtOH, our mouse study used forced EtOH exposure via vapor inhalation. It is also possible that in genetically heterogenous human populations, genetic influences on drinking behavior mask the epigenetic effects of paternal EtOH exposure. Lastly, it is also important to keep in mind that in rodents it is impossible to model all aspects of excessive human drinking with a single drinking assay. In the current mouse study, EtOH drinking was only tested using a single test, the two-bottle, free choice assay. It is conceivable that other behavioral tests of rodent drinking behavior such as those that model binge type drinking (e.g., drinking in the dark) or reward based drinking (e.g., operant conditioning) may be more relevant in the current context to human drinking.

While our drinking data are in conflict with human studies, paternal EtOH exposure also increased sensitivity to the anxiolytic and motor enhancing effects of EtOH in male offspring. In humans, increased sensitivity to the subjective effects of EtOH is associated with decreased risk of developing alcoholism [43]. Notably, in our study, increased sensitivity to EtOH in E-sired male offspring was associated with decreased EtOH consumption. Heritability of EtOH sensitivity on motor tests has also been noted in humans. Of note, static ataxia (body sway) after EtOH consumption shows heritability and is associated with a sexually dimorphic pattern of inheritance [44]. Compared to C-sired male offspring, E-sired males were more sensitive to low dose EtOH enhancement of motor coordination on the accelerating rotarod assay and to locomotor stimulation (and anxiolysis) on the elevated plus maze but not on the open field assay. The discrepancy between the elevated plus maze and open field locomotor results was likely due to the EtOH dose and timing, since several studies have reported 1 g/kg EtOH did not induce changes in locomotion on the open field assay [45]–[47]. It is also notable that E-sired male offspring demonstrated subtle changes on the accelerating rotarod assay, with only the 5^th^ trial reaching statistical significance compared to C-sired male offspring. This finding is more suggestive of an effect on motor learning than locomotor enhancement. These issues raise the possibility that paternal EtOH exposure affects discrete pathways to alter sensitivity to EtOH and we anticipate future studies to more fully characterize EtOH-induced behaviors in offspring. Our study also showed no effect of paternal EtOH exposure on EtOH metabolism in offspring, which is consistent with human studies of children of alcoholics [48]. These findings strongly suggest that behavioral differences in E-sired offspring are being driven by neurobiological changes that alter sensitivity to EtOH to decrease drinking.

EtOH exposure induced hypomethylation at both loci studied in motile sperm and hypomethylation at one of these, the *Bdnf* exon IXa promoter, was maintained in the VTA of E-sired offspring. This finding is consistent with studies that show both maternal and paternal preconception EtOH exposure alter DNA methylation at imprinted loci in offspring [49], [50]. While EtOH's effects on DNA methylation in offspring are striking, EtOH is known to alter several epigenetic marks across tissue types and could act as a broader epimutagen in sperm. For example, EtOH alters histone modifications in the amygdala, which contribute to its acute anxiolytic effects and withdrawal-induced anxiety [51], [52]. miRNA regulation by EtOH has been shown to underlie changes in expression of BK channel splice variants [53]. Therefore, it is conceivable that EtOH induces multiple heritable epigenetic modifications in germ cells and these marks are maintained in the brain of offspring sired by alcohol exposed fathers.

This idea is especially intriguing considering epigenetic reprogramming during spermatogenesis is highly plastic. These changes include chromatin compaction by replacement of most histones with protamines, de novo DNA methylation and maintenance, and silencing of retrotransposable elements through numerous small regulatory RNAs [54]. As discussed previously, EtOH acts as an epimutagen in other tissues and may be affecting multiple epigenetic processes during spermatogenesis. A rodent study demonstrated chronic EtOH exposure decreases cytosine methyltransferase levels in the testes [55], which is consistent with our observation of decreased DNA methylation at the IG DMR and *Bdnf* exon IXa promoter. A recent human study also demonstrated that EtOH consumption was correlated with decreased methylation of imprinted genes that are normally hypermethylated in human sperm [56]. EtOH's effects on DNA methylation in gametes are further supported by the observation that chronic EtOH alters methionine metabolism [57], which is critically involved in the function of cytosine methyltransferases [58]. We expect future studies to expand on DNA methylation as well as begin to study EtOH-induced changes to retained histones and noncoding RNAs.

In conclusion, upon paternal EtOH exposure, EtOH likely functions as an epimutagen imparting long lasting effects that ultimately impact the next generation. Prior rodent studies demonstrated an impact of paternal EtOH exposure on brain development and numerous basal behaviors. The results presented here demonstrate an effect on behavioral sensitivity to EtOH, EtOH drinking behavior, and gene expression that is restricted to male offspring. If these rodent studies apply to humans drinking alcohol, the results have far reaching implications considering the large percentage of the human population that consume alcohol prior to procreation.

## Supporting Information

Figure S1 **A subset of offspring was tested for their preference for saccharin or quinine vs. water.** There were no significant differences between E- (n = 11) and C-sired (n = 10) male offspring on (A) quinine drinking or (B) saccharin drinking; there were also no significant differences between E- (n = 6) and C-sired (n = 5) female offspring on (C) quinine drinking or (D) saccharin drinking. Data presented as mean ± SEM.
(TIF)

Figure S2 **Offspring were tested for locomotor activity in an open field 20 minutes after i.p. injection of 1 g/kg EtOH or saline.** There were no significant differences among (A) E- and C-sired male offspring or (B) female offspring after treatment with EtOH. n = 7–8/group. Data presented as mean ± SEM.
(TIF)

Figure S3 **EtOH metabolism was measured after i.p. injection of 3.5 g/kg EtOH in saline.** There were no significant differences in blood EtOH levels 60 minutes and 240 minutes after EtOH treatment between E- and C-sired (A) male or (B) female offspring. n = 4–5/group. Data presented as mean ± SEM.
(TIF)

Figure S4 **Expression of *Bdnf* exons IV and IXa were measured in the medial prefrontal cortex (mPFC) of offspring.** There were no significant differences between (A) male and (B) female E- and C-sired offspring in expression of *Bdnf* exons IV and IXa.
(TIF)

Figure S5 **Full bisulfite sequencing results represented in Fig. 6C–E.** Each circle represents one of the 17 potentially methylated cytosines in the *Bdnf* exon IXa promoter; filled circles are methylated and unfilled circles are unmethylated. Each block of rows represents sequenced colonies from a single independent animal. n = 4–7/group.
(TIF)

Figure S6 **We measured DNA methylation at the intergenic (IG) differentially methylated region (DMR) in motile sperm using bisulfite sequencing.** (A) DNA methylation is significantly reduced at the IG DMR in motile sperm of EtOH-exposed sires relative to room air controls. (B) Quantification of bisulfite sequencing results. Each circle represents one of the 33 potentially methylated cytosines in the IG DMR; filled circles are methylated and unfilled circles are unmethylated. Each block of rows represents sequenced colonies from a single independent animal. n = 6–7/group. Data presented as mean ± SEM. *p<0.0001.
(TIF)
