# Supplementary figures and images for: Paternal Alcohol Exposure Reduces Alcohol Drinking and Increases Behavioral Sensitivity to Alcohol Selectively in Male Offspring
Source: PLoS One. 2014 Jun 4;9(6):e99078. doi: 10.1371/journal.pone.0099078 (PMC4045990; doi:10.1371/journal.pone.0099078)

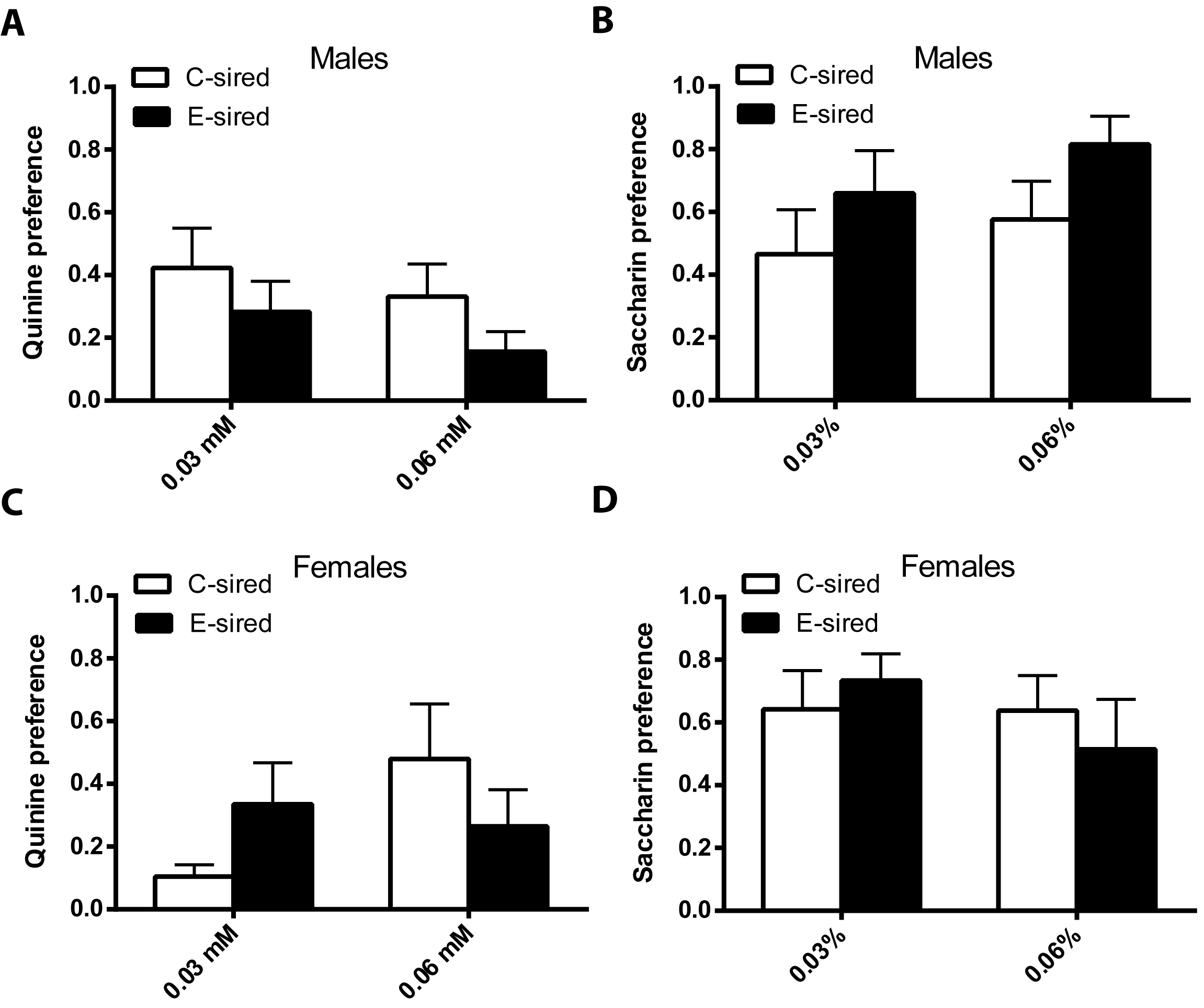

Supplement: Figure S1 — A subset of offspring was tested for their preference for saccharin or quinine vs. water. There were no significant differences between E- (n = 11) and C-sired (n = 10) male offspring on (A) quinine drinking or (B) saccharin drinking; there were also no significant differences between E- (n = 6) and C-sired (n = 5) female offspring on (C) quinine drinking or (D) saccharin drinking. Data presented as mean ± SEM. (TIF) [file pone.0099078.s001.tif]

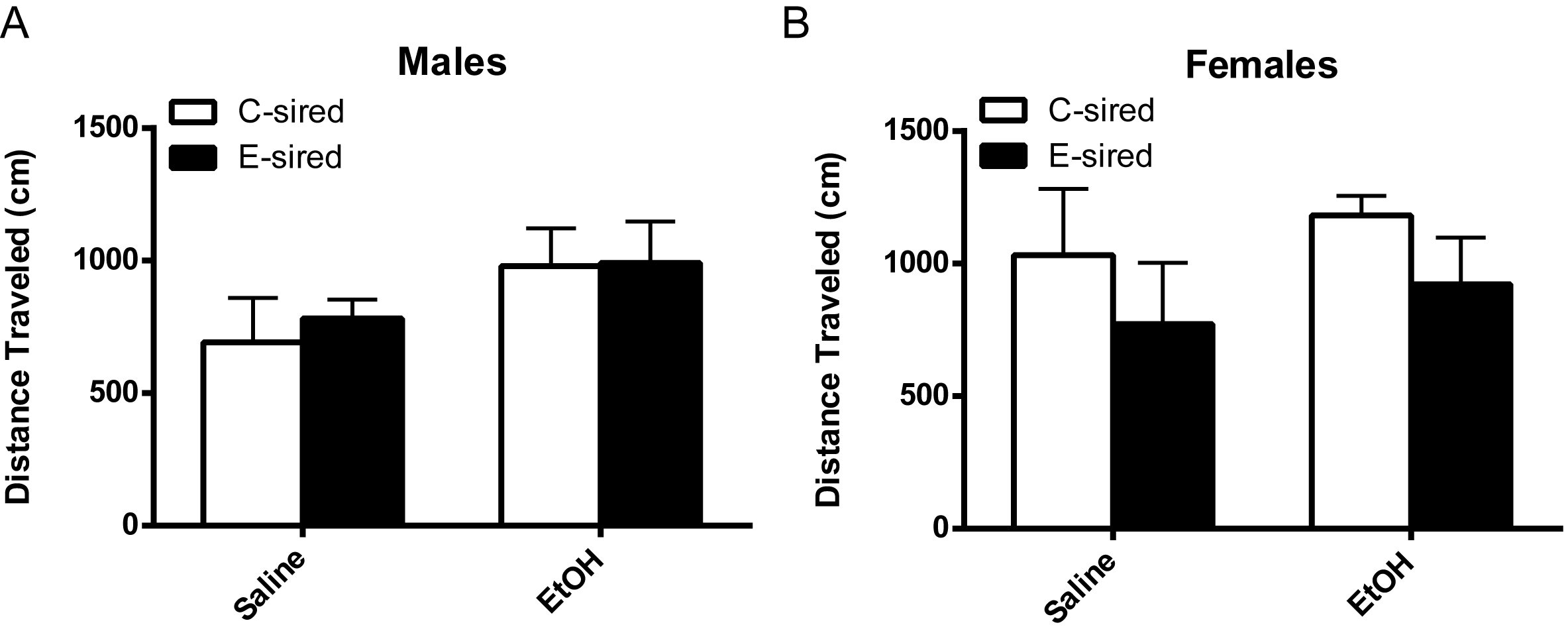

Supplement: Figure S2 — Offspring were tested for locomotor activity in an open field 20 minutes after i.p. injection of 1 g/kg EtOH or saline. There were no significant differences among (A) E- and C-sired male offspring or (B) female offspring after treatment with EtOH. n = 7–8/group. Data presented as mean ± SEM. (TIF) [file pone.0099078.s002.tif]

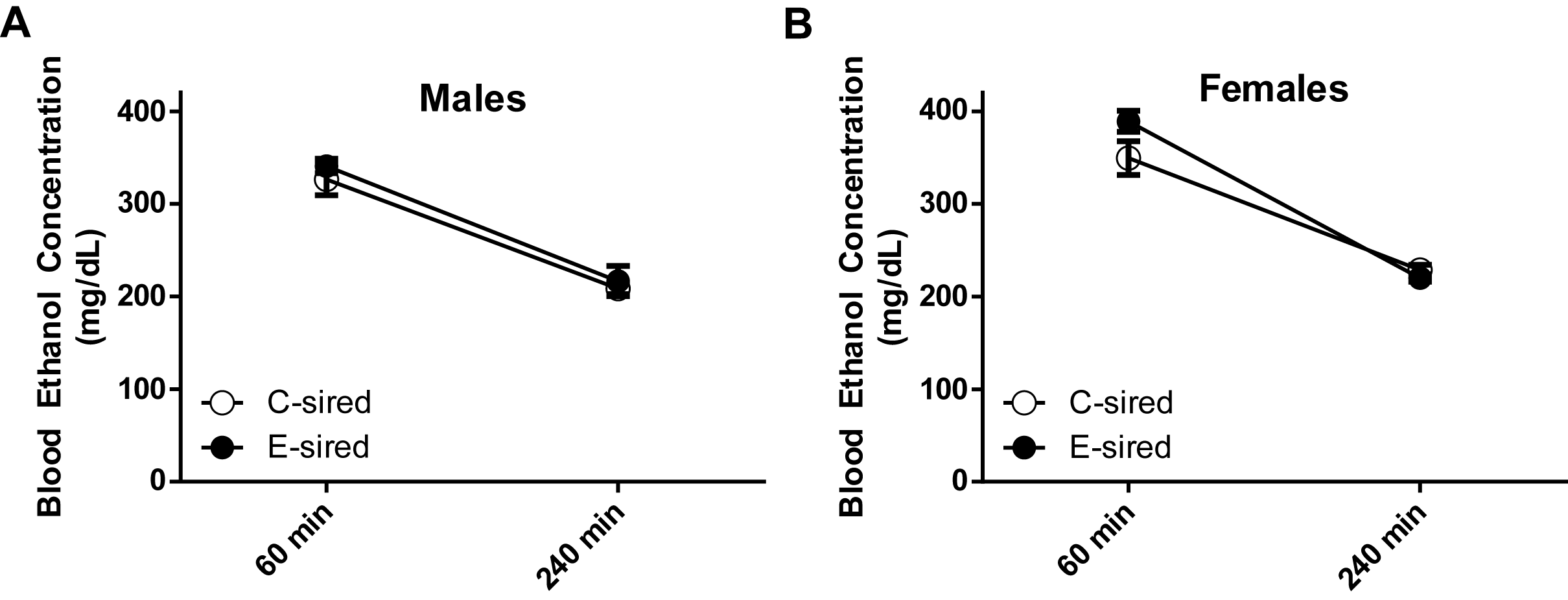

Supplement: Figure S3 — EtOH metabolism was measured after i.p. injection of 3.5 g/kg EtOH in saline. There were no significant differences in blood EtOH levels 60 minutes and 240 minutes after EtOH treatment between E- and C-sired (A) male or (B) female offspring. n = 4–5/group. Data presented as mean ± SEM. (TIF) [file pone.0099078.s003.tif]

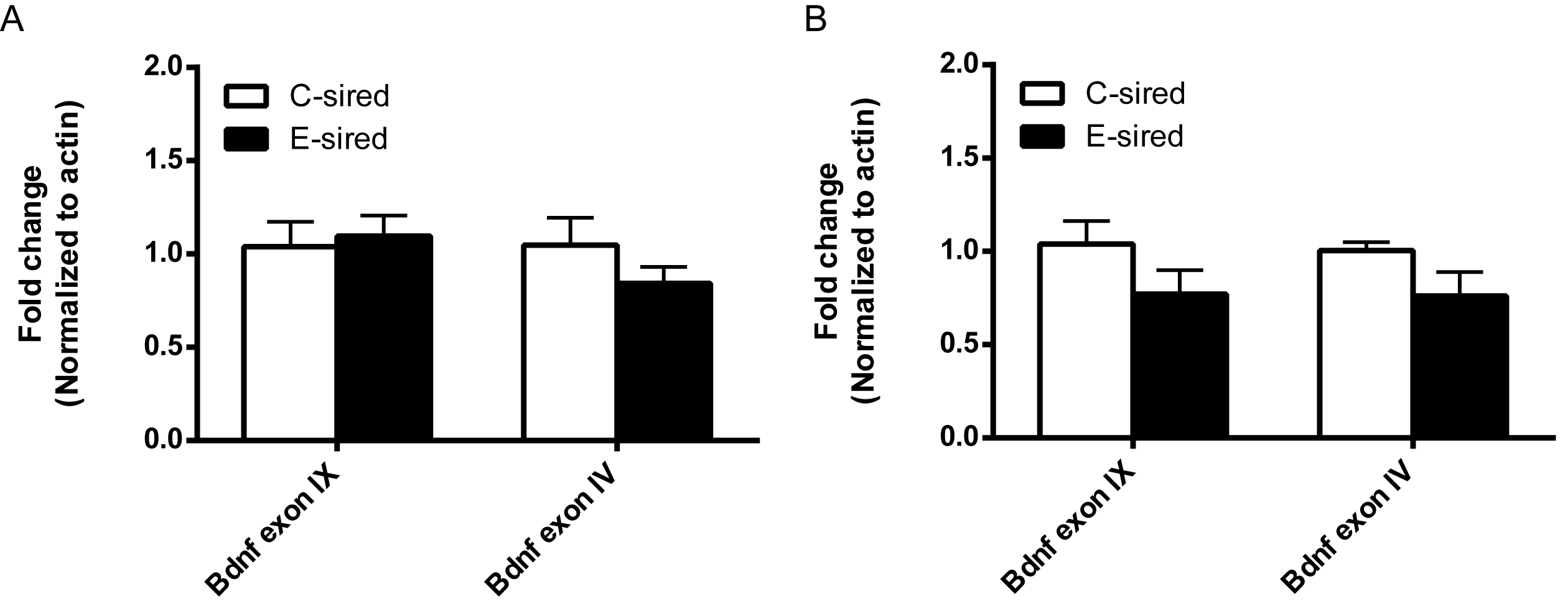

Supplement: Figure S4 — Expression of Bdnf exons IV and IXa were measured in the medial prefrontal cortex (mPFC) of offspring. There were no significant differences between (A) male and (B) female E- and C-sired offspring in expression of Bdnf exons IV and IXa. (TIF) [file pone.0099078.s004.tif]

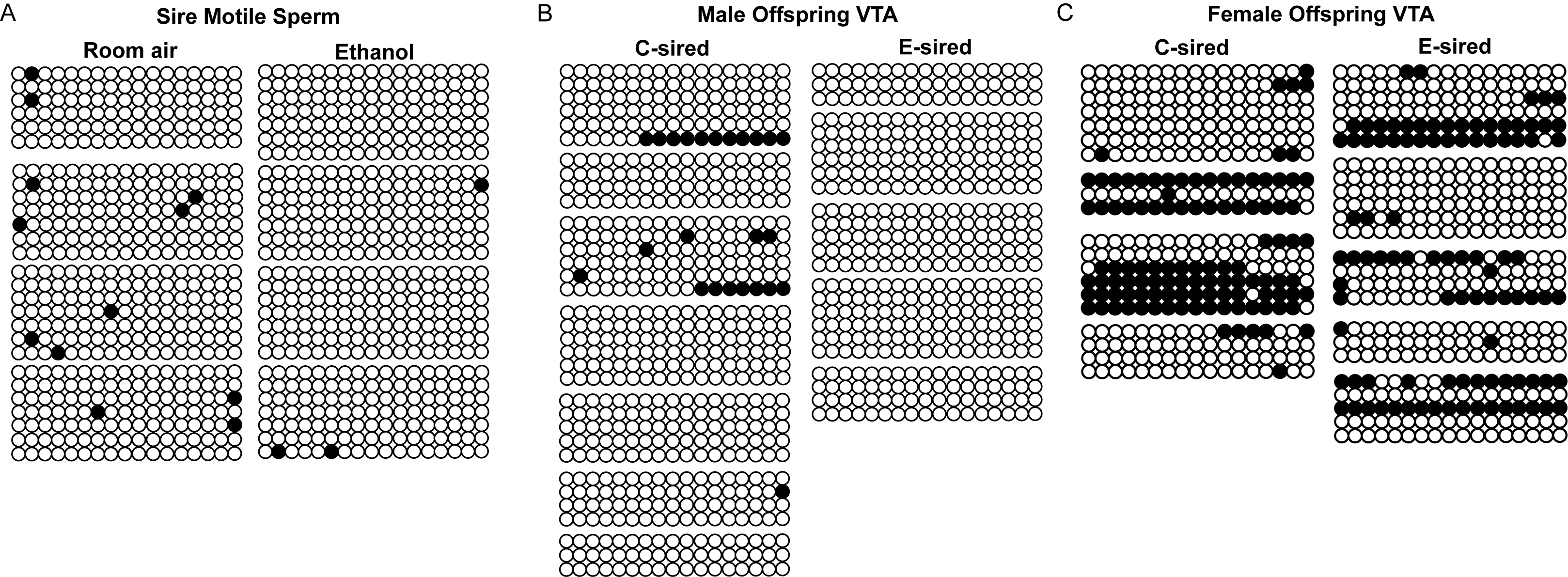

Supplement: Figure S5 — Full bisulfite sequencing results represented in Fig. 6C–E. Each circle represents one of the 17 potentially methylated cytosines in the Bdnf exon IXa promoter; filled circles are methylated and unfilled circles are unmethylated. Each block of rows represents sequenced colonies from a single independent animal. n = 4–7/group. (TIF) [file pone.0099078.s005.tif]

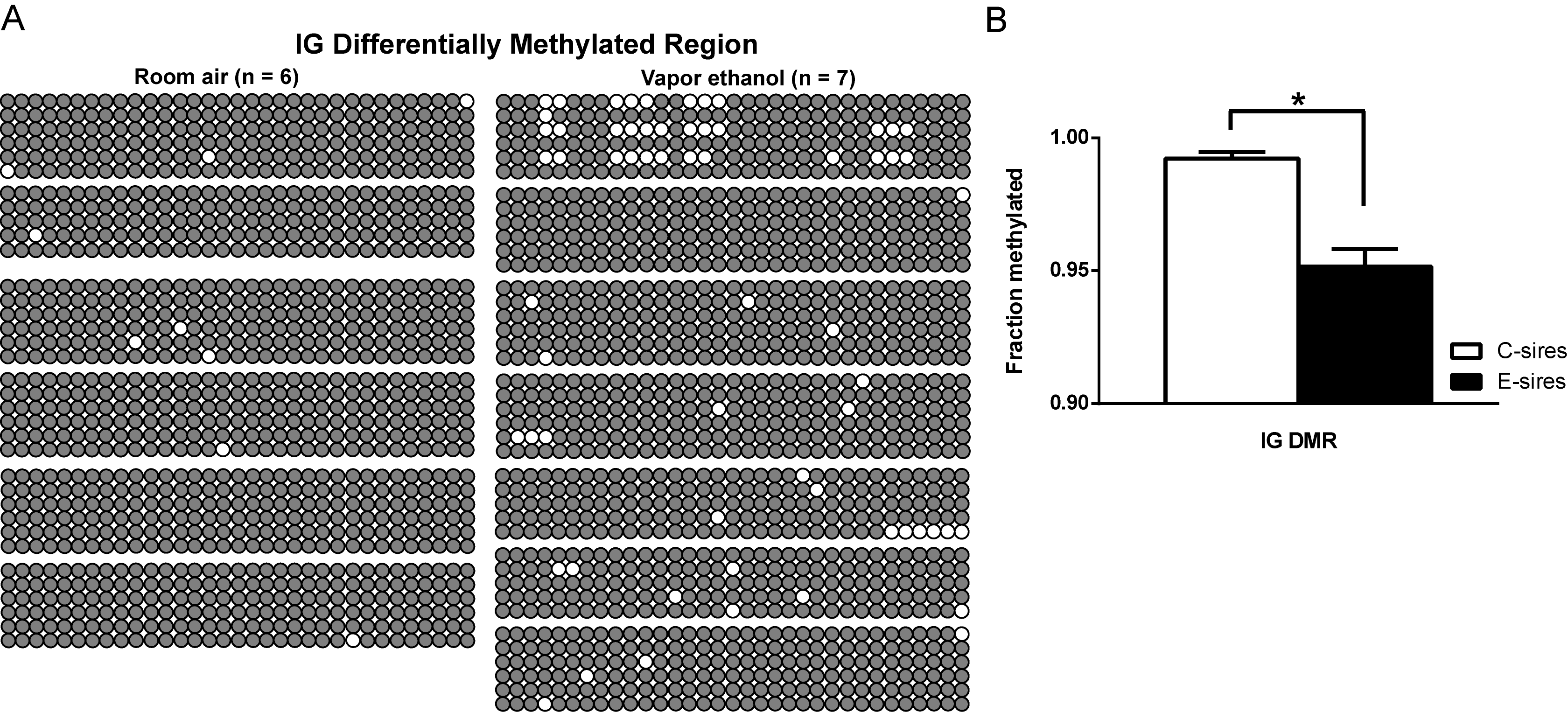

Supplement: Figure S6 — We measured DNA methylation at the intergenic (IG) differentially methylated region (DMR) in motile sperm using bisulfite sequencing. (A) DNA methylation is significantly reduced at the IG DMR in motile sperm of EtOH-exposed sires relative to room air controls. (B) Quantification of bisulfite sequencing results. Each circle represents one of the 33 potentially methylated cytosines in the IG DMR; filled circles are methylated and unfilled circles are unmethylated. Each block of rows represents sequenced colonies from a single independent animal. n = 6–7/group. Data presented as mean ± SEM. *p<0.0001. (TIF) [file pone.0099078.s006.tif]
